# Centenarians Today: New Insights on Selection from the 5-COOP Study

**DOI:** 10.1155/2010/120354

**Published:** 2011-03-07

**Authors:** Jean-Marie Robine, Siu Lan Karen Cheung, Yasuhiko Saito, Bernard Jeune, Marti G. Parker, François R. Herrmann

**Affiliations:** ^1^National Institute on Health and Medical Research, INSERM, 75654 Paris, France; ^2^Department of Social Work and Social Administration, The University of Hong Kong, Hong Kong; ^3^Nihon University Advanced Research Institute for the Sciences and Humanities, Tokyo 102-8251, Japan; ^4^Danish Aging Research Center, Institute of Public Health, University of Southern Denmark, 5000 Odense, Denmark; ^5^Aging Research Center, Karolinska Institute, Stockholm University, 113 30 Stockholm, Sweden; ^6^Department of Rehabilitation and Geriatrics, Geneva University Hospitals and University of Geneva, 1226 Thônex Geneva, Switzerland

## Abstract

The number of oldest old grew tremendously over the past few decades. However, recent studies have disclosed that the pace of increase strongly varies among countries. The present study aims to specify the level of mortality selection among the nonagenarians and centenarians living currently in five low mortality countries, Denmark, France, Japan, Switzerland, and Sweden, part of the 5-Country Oldest Old Project (5-COOP). All data come from the Human Mortality Database, except for the number of centenarians living in Japan. We disclosed three levels of mortality selection, a milder level in Japan, a stronger level in Denmark and Sweden and an intermediary level in France and Switzerland. These divergences offer an opportunity to study the existence of a trade-off between the level of mortality selection and the functional health status of the oldest old survivors which will be seized by the 5-COOP project.

## 1. Introduction

The number of very old people greatly increased during the past few decades, for instance, the number of centenarians (100+) increased from 154 people in Japan in 1963 when the first *centenarian list* (*Zenkoku koureisha meibo*) was released by the Ministry of Health and Welfare [1] to 40,399 people in 2009 [2]. In France, this number also increased from a few hundred in 1950 to 14,944 by 2010 [3]. The first study assessing the emergence of the centenarians at the global level, in the mid 1990s, concluded that their number doubled every 10 years since the 1960s in the low mortality countries [4]. However, recent studies disclosed that the pace of increase in the number of oldest old strongly varies among the developed countries which are no longer in a phase of convergence regarding their mortality conditions [5]. Indeed during the 10-year period, from 1996 to 2006, the number of centenarians was multiplied by 4 in Japan while it was only multiplied by 2 in Europe [6]. Among the 27 European countries studied, this increase varied from a factor higher than 2 in some countries such as Austria, Italy, Germany, and Spain to a factor lower than 1.5 in Nordic or Eastern European countries such as Norway, Iceland, Latvia, Lithuania or Bulgaria. 

During the same time various reports from Japan suggested that the functional health status of the centenarian people strongly decreased, especially their mobility with a higher proportion of people bedridden or confined to their house, as their number increased [5, 7, 8], while Danish studies suggested a *statu quo *(cognitive scores), or even an improvement for females (self reported ADLs), in the functional health status of the centenarians living in Denmark [9, 10]. In absence of reports examining the change over time in the functional health status of the centenarians in other countries, these Japanese and Danish studies suggest the possible existence of a trade-off between the proportion of people reaching the age of 100 and the functional health status of the survivors. Indeed the number of centenarians was only multiplied by 1.6 in Denmark between 1996 and 2006 (1.3 for males and 1.7 for females) versus 4.2 in Japan (3.0 for males and 4.5 for females) [6]. 

The trade-off between two characteristics, such as fertility and longevity, is not an uncommon phenomenon in biological science. Similar kinds of trade-offs seem to also exist in the social sciences, such as that between the quantity and the quality of goods. In population health science, this has translated into the ongoing debate about the quantity and quality of years lived and led to the development of summary measures of population health, such as health expectancies, in order to assess whether an increase in life expectancy (quantity of life) is accompanied by an equivalent increase in the quality of the years lived [11]. The existing theories, “compression of morbidity” [12], “pandemic of disability” [13, 14], and “dynamic equilibrium” [15], illustrate all possible combinations between the changes in the quantity and quality of years lived. They do not provide a theory on the relationship between the level of mortality selection (how easy or difficult it is to survive to a given age) and the functional health status of the survivors (i.e., the people reaching this given age). It is generally thought that it is the frail who succumb first, leaving alive the more robust. It has also been suggested that this selection may be strong enough at the highest ages to virtually stop mortality rates from rising, and even to result in a decline [16–18]. Kannisto has suggested that this selection process may impair the health of some survivors leaving them frailer. Thus when the selection process is stronger, causing extra deaths, the health of many survivors is impaired [19]. Under these conditions it seems difficult to predict the impact of the level of mortality selection on the health status of survivors. A stronger selection may only keep alive the most robust individuals but the selective process itself may have impaired them, while a milder selection may keep alive less robust persons, but without having resulted in a deterioration of their health. 

The current divergence in the fall of mortality above age 80 observed among the low mortality countries, and therefore in the level of selection of the survivors at age 100, offers an opportunity to study the existence of such a trade-off between the level of mortality selection and the functional health status of the oldest old survivors. This opportunity has been seized by the 5-COOP project (5-Country Oldest Old Project) which aims to accurately compare the health status of centenarians living in Denmark, France, Japan, Sweden, and Switzerland. Although the 5-COOP project will focus on the cohorts of people born in 1911 (and later) who will reach their 100th birthday from 2011 onwards, this paper details the differences in mortality selection among the five countries using the mortality history of the 1905 and 1910 cohorts.

The results are presented in three sections. After Section 3.1 quickly reviews the centenarian figures, Section 3.2 describes the mortality experiences of the male and female 1905 birth cohorts, from age 50 in 1955 to age 100 in 2005, as well as the mortality experiences of the 1910 birth cohorts, from age 50 in 1960 to age 95 in 2005.  Section 3.3 presents the results in terms of selection from the 80th to the 100th birthdates before the significance of the observed differences in mortality selection is discussed.

This paper does not provide direct information on the health status of the people currently reaching their 95th or 100th year in the 5 countries of the study but merely describes their mortality experience from ages 50 to ages 95 and 100, respectively. 

## 2. Data

All data come from the Human Mortality Database—HMD (http://www.mortality.org/), except for the number of centenarians in Japan, and were downloaded in Spring 2009 for the number of centenarians and in Spring 2010 for the death rates. The numbers of centenarians used in Section 3.1 are estimated by January first of each year (HMD, period data, population size), except in Japan where a list of living centenarians provided annually by the Ministry of Health and Welfare has been used. The Japanese counts are by September 30th. The death rates used in Sections 3.2 and 3.3 are cohort data coming without exception from the HMD (HMD, cohort data, death rates). The centenarian terminology is vague. Therefore, it is specified in the paper when the number of centenarians (100) corresponds to the people in the single age 100 and when the number of centenarians (100+) corresponds to the people aged of 100 years and over.

## 3. Results

### 3.1. The Centenarian Figures in the Five Countries

Since 1946, the number of centenarians increased tremendously in the five countries under study. However a quick look at the graphs listed in Supplementary Material (Figure A1) discloses significant differences in this increase. If it looks exponential in France, it seems to be much more rapid in Japan and conversely almost linear in Denmark.  Table 1 summarizes this information over the last decade preceding 2006. Beyond the size effect, the Japanese population, being about twofold the size of the French population, and the latter being about ten times the size of the Swiss population, it is clear that the pace of increase of the number of centenarians is not similar in the five countries. It is much more rapid in Japan and, comparatively, quite slow in Denmark and Sweden, especially for males (Table 1). 

 The 10-year increase factor, 1996–2006 for both sexes, varies from 4.2 in Japan to 1.6 in Denmark (2.0 in France, 1.9 in Switzerland and 1.7 in Sweden). The centenarian population is predominantly a female population with a sex-ratio close to six women for every man. Among the five countries, it is at a maximum in France with a ratio of 7.1 in 2006 and minimum in Switzerland with a ratio of 5.3.

These differences in the pace of increase in the number of centenarians may be caused by two main factors, that is, a differential in population growth and/or a differential in mortality selection. The differential in population growth, and especially the differential in birth cohort growth, clearly contributed to the differences in the 10-year increase factor between Japan and the four European countries. Indeed, in the last quarter of the 19th century, the birth cohort size significantly increased in Japan, moving from 869,126 new born in 1875 to 1,420,534 new born in 1900. Although quite important, such a growth cannot explain, by far, the observed differences in the increase in the number of centenarians. The following sections explore the second mechanism, that is, the differential in mortality selection and its impact on the number of centenarians, beginning at age 50.

### 3.2. The Mortality Experiences of the 1905 and 1910 Birth Cohorts

The two cohorts, born in 1905 and 1910, have been selected both for several practical and analytical purposes: (i) availability of population and mortality data in the five countries studied until ages 95 and 100, which are the ages of interest in the 5-COOP project, (ii) proximity to the cohorts which will be involved in 5-COOP, born in 1911 and later, and (iii) correspondence with the study of the Danish nonagenarian and centenarian cohorts [9, 10, 20–22]. 

**Figure 1:**
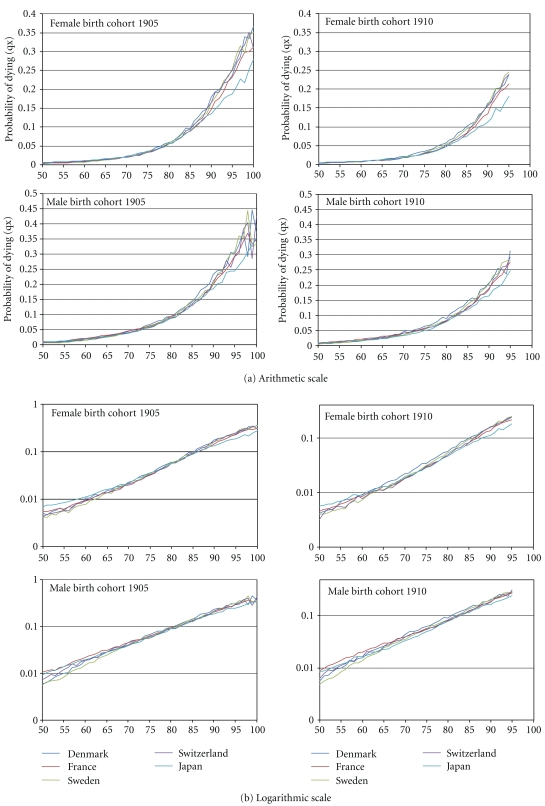
Mortality trajectory with age, from age 50, in the cohorts born in 1905 and 1910: Denmark, France, Japan, Sweden, and Switzerland.

The mortality experiences of the Japanese cohorts born 1905 and 1910 display a lower age mortality trajectory above the age of 85 years compared to the four European countries in the study. In detail (see Figures 1(a) and 1(b)), the Japanese female cohort born in 1905 experienced a higher level of mortality than the European cohorts of our study from age 50, reached in 1955, to age 66, reached in 1971. Then, the morality rates of the various countries overlap until age 90, reached in 1995. From this age to the age of 100 years the Japanese cohort experienced the lowest level of mortality. The Japanese cohort still experienced several times the highest mortality level between ages 66 and 75, and yet several times the lowest mortality level between age 75 and 90. The last time this Japanese cohort experienced the highest mortality level was in 1980, at the age of 75 years, and the first time it experienced the lowest mortality level was in 1985 at the age of 80 years. Compared to this main difference, differences among European cohorts seem to be quite small, though the French cohort seems to have experienced an intermediary situation with a much larger overlap with the other European cohorts.

The second Japanese female cohort born in 1910 experienced a higher level of mortality than the European cohorts of our study from age 50, reached in 1960, to age 59, reached in 1969, with the exception of age 56. Then again the mortality rates of the various cohorts overlap until age 85, reached in 1995. From this year, where the cohort has reached its 85th birthday, this second Japanese cohort also experienced the lowest level of mortality. The 1910 Japanese cohort still experienced several times the highest mortality level between ages 59 and 64, and yet several times the lowest mortality level before age 85. The last time this Japanese cohort experienced the highest mortality level was in 1974, at age 64, and the first time it experienced the lowest mortality level was in 1984, at age 74.

 Except in 1960 at the age of 55 years, the Japanese male cohort born in 1905 never experienced a higher level of mortality than the European cohorts of our study. The French male cohort experienced the highest mortality level from age 50 to age 70, reached in 1975, age 55 excepted. Conversely, from age 69 (in 1974) to age 100, the Japanese cohort experienced the lowest level of mortality, if we make exceptions for a few ages, that is, ages 75, 76, 82, 85–87, 89, 97–100. Although overlapping for a few years with other trajectories, the Japanese mortality trajectory with age, above the age of 70 years, is clearly lower than the European trajectories. On the other hand, the Swedish male cohort experienced the lowest mortality level from age 50 to age 68 (in 1973), with the exception of age 51, but experienced some of the highest mortality levels at age 95 and above (ages 95, 96 and 98) from the year 2000 and on.

The Japanese male cohort born in 1910 also never experienced a higher level of mortality than the European countries of our study. The French male cohort experienced the highest mortality level from age 50 to age 65, reached in 1975. Before age 65, reached in 1975, the Swedish cohort experienced the lowest level of mortality. At age 65 and above the Japanese cohort experienced the lowest level of mortality, if we exclude ages 82 and 84, ages at which the French cohort experienced the lowest mortality levels. Thus, the Japanese mortality trajectory with age is clearly lower than the European trajectories above age 65. The Swedish male cohort born in 1910 also experienced the lowest mortality level before age 65 and some of the highest above age 90 (ages 91, 93 and 94).

Beyond the most spectacular differences (i.e., the female Japanese cohorts experiencing the highest mortality levels before the 1970s and the lowest after the mid 1990s, or the male Swedish cohorts experiencing the lowest mortality levels before the mid 1970s and some of highest levels since the year 2000), and some similarities between genders within the same country, the mortality experiences are clearly country and gender specific. The Danish cohorts often, but not always, experienced the highest morality levels since the 1970s for females and since the mid 1970s for males. The Swiss female cohorts regularly experienced the lowest mortality in the 1970s and 1980s while the 1910 Swiss male cohorts never experienced the highest or the lowest mortality levels. Contrary to the male cohorts, the French female cohorts never experienced the highest mortality levels. On the contrary they experienced some of the lowest levels at the beginning of the 1990s. These country-specific mortality experiences led to different levels of selection for the cohort members who reached their 95th birthday (birth cohort 1910) or 100th birthday (birth cohort 1905) during the calendar year 2005 and which are detailed in the following section.

The countries experiencing the lowest and the highest mortality levels by single age, for each gender and each cohort, through the age mortality trajectory above the age of 50 years, are reported in Supplementary Material (Table A1). The diagonal arrangement of the lowest and highest mortality levels in this Table suggests a strong period effect, the reasons for which will be debated in the discussion.

### 3.3. Selection from Age 80 to Age 100

**Figure 2:**
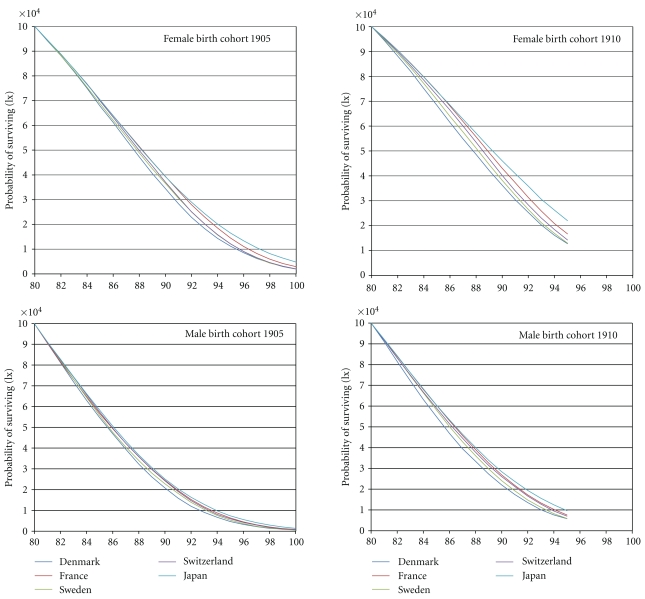
Survival curves from age 80, for the cohorts born in 1905 and 1910: Denmark, France, Japan, Sweden, and Switzerland.

The mortality selection process which occurred in the five countries above the age of 80 years is represented on Figure 2.

For the female cohorts born in 1905 and for 100,000 survivors at age 80, it led to very similar numbers of individuals surviving to age 100 in Denmark, Sweden, and Switzerland (1,957, 2,014 and 2,022 people, resp.), a noticeably higher number in France (2,928) and a strikingly higher number in Japan (4,780). This offers three levels of mortality selection: stronger (Denmark, Sweden, and Switzerland), milder (Japan) and intermediate (France).

For the male cohorts born in 1905, the mortality selection from age 80 led to low numbers of individuals surviving to 100 in Denmark and Sweden (532 and 570, resp.), noticeably higher numbers in Switzerland and France (776 and 856, resp.) and a strikingly higher number in Japan (1,376). This offers again three levels of mortality selection: stronger (Denmark and Sweden), milder (Japan) and intermediate (France and Switzerland).

 For the female cohorts born in 1910 (for 100,000 survivors at age 80), the mortality selection led to similar numbers of individuals surviving to age 95 in Denmark and Sweden (12,709 and 12,923, resp.), noticeably higher numbers in Switzerland (14,241) and France (16,668) and a strikingly higher number in Japan (21,974). This offers three or four levels of mortality selection: stronger (Denmark and Sweden), milder (Japan) and intermediate (France), with Switzerland between France, on the one hand, and Denmark and Sweden, on the other hand.

For the male cohorts born in 1910, the mortality selection from age 80 led also to similar numbers of individuals surviving at age 95 in Denmark and Sweden (5,760 and 5,893, resp.), noticeably higher numbers in Switzerland (6,995) and France (7,593) and a strikingly higher number in Japan (9,622), offering three or four levels of mortality selection: stronger (Denmark and Sweden), milder (Japan) and intermediate (France) with Switzerland between France and both Denmark and Sweden.

## 4. Discussion

During the decade, 1996–2006, the number of centenarians in Japan increased two to three times faster compared with Denmark and Sweden, and two times faster when compared to Switzerland and France. The strong increase in the size of the Japanese birth cohorts at the end of the 19th century explained only a small part of the gaps. The main factor which explains the observed differences in the pace of increase in the numbers of oldest old in the five countries studied is the differential in mortality selection at older ages. Indeed, compared to Danish women, it has been 2.4 times easier for Japanese women born in 1905, and still alive at age 80 in 1985 to become a centenarian in 2005 (2.6 times easier for the male cohort) and 1.7 times easier for Japanese men and women born in 1910 and still alive at age 80 in 1990 to reach the age of 95 years in 2005 (see Table 2).

We studied the mortality experience of the various birth cohorts beginning at age 50. This does not mean that the mortality experience before age 50 does not matter in determining the number of people surviving to age 100 [23, 24], but in this study we were more interested in the mortality selection among adult people than in knowing how many persons in a certain birth cohort became centenarians. We are especially interested in the mortality selection among the oldest old. This is the reason why the last section focused on age 80 and above only. In a study of the demography of centenarians in England and Wales, it has been demonstrated that improved survival from age 80 to age 100 explained at least two times more the increase in the total numbers of centenarians (100+) than improved survival from birth to age 80 for the cohorts born between 1851 and 1896 [23]. 

The 1905 birth cohorts reached 50 years of age in 1955, ten years after World War II. The 20th century was not a tranquil century. The cohort born in 1905 and 1910 faced wars in Asia and in Europe and severe economic crises in their youth. The involvement of these cohorts in World War I and II varied strongly according to country, year of birth, and gender. Switzerland, for instance, escaped much of the devastation of the wars while France and Japan suffered both loss of manpower and destruction of property. Also, infant mortality was high in both 1905 and 1910 and stood at different levels in the five countries. However, recent studies demonstrated the preponderance of period factors to explain changes in mortality level, especially among the oldest old, even if early life and middle life factors also contribute to old age survival and mortality [25]. Nevertheless, ignoring mortality before age 50 is a limitation of this study. 

Although the mortality experiences starting at age 50 were specific for each cohort, our study disclosed strong period effects punctuating these experiences. For instance, among cohorts born in 1905 and 1910, Japanese females experienced the highest level of mortality before 1970 and the lowest after 1995. Similarly, the French male cohorts experienced the highest mortality level before 1975 and the Swedish male cohorts the lowest. These same Swedish cohorts experienced some of the highest mortality levels after 2000. In all these cases, the calendar year appears more important than the year of birth. This helps us to generalize the mortality experience of the oldest old of the five countries. 

In summary, our study disclosed three levels of mortality selection among the centenarians currently living in the five countries.

- *A milder level of selection in Japan*. Indicated by the fact that Japanese women who became centenarians in 2005 had a 2.4 times higher chance to survive from age 80 to 100 than their Danish counterparts (2.6 times higher chance for the male cohort). The better mortality conditions observed in Japan are a recent phenomenon. Indeed before 1970 Japanese women experienced the highest level of mortality and crossed over the mortality trajectories of the other countries in the 1970s and 1980s.
- *A stronger level of selection in Denmark and Sweden*. Denmark and Sweden, almost experienced the opposite mortality changes than Japan. Thus the worse mortality conditions mainly observed today in Denmark, and secondarily in Sweden, are also recent phenomena. In particular, before 1975, Swedish cohorts experienced the lowest level of mortality.
- *An intermediary level of selection in France and Switzerland*. Indicated by the fact that the French (males and females) and Swiss males who became centenarians in 2005 had about a 1.5 times higher chance to survive from age 80 to 100 compared to their Danish counterparts—French men experienced similar mortality changes to those experienced by Japanese females. Indeed, before 1975, French males experienced the highest mortality level while they are second to Japan for the most recent years. On their side, the Swiss cohorts underwent specific changes over the same period of time. In particular the Swiss female cohorts regularly experienced the lowest mortality in the 1970s and 1980s.

These various epidemiologic histories have determined the current levels of mortality selection met by the centenarians living today in Denmark, France, Japan, Switzerland and Sweden. Three levels have been identified, milder, stronger and intermediary. However, due to the lack of studies or the lack of comparative studies, it is impossible to assess whether these different levels of selection have had an impact on the health status of the survivors. On one side reports have suggested a worsening in the functional health status of centenarians living in Japan over time [7, 8]. On another side reports have suggested a *status quo *(cognitive scores) and even an improvement for women (self reported ADLs), in the functional health status of centenarians living in Denmark [9, 10]. Although there are no similar reports on the health status of centenarians in Sweden, the Swedish Panel Study of the Living Conditions of the Oldest Old (SWEOLD) suggests some deterioration in the health status of the oldest old in the recent years [26, 27]. In France as well as in Switzerland, there is very little available information on the health status of the oldest old. The Danish 1905 cohort study is clearly in favor of the mainstream hypothesis that it is the frail who succumb first, strongly limiting the increase of the prevalence of geriatric conditions, such as ADL disability or dementia, with age [22]. 

It is worth reminding that some conditions have an effect on both disability and mortality, such as obesity and cardiovascular diseases, some on disability only, such as arthritis, macular degeneration or mild dementia, and some mainly on mortality, such as cancer [28, 29]. In addition to these various health outcomes specific for each disease, the possible role of the balance between life-saving medical interventions, rehabilitation of disabling conditions, and prevention of both disabling and lethal conditions must be mentioned. If a country mostly invests in life-saving interventions, disregarding the disabling conditions, the increased survival will be likely accompanied by an increase in the prevalence of disability. Different health policies, especially focusing on health behaviors such as alcohol and tobacco consumption, can entail survivorships with different functional health status. The characteristics of the daily care, formal and informal, provided to the oldest old, should also be at play. Can differences in survival between Europe and Japan or within European countries be explained by such factors? 

It is in this context that the 5-Country Oldest Old Project (5-COOP) aims to provide prevalence estimates for a series of bio-markers, functional limitations, and geriatric conditions, including dementia and cognitive disorders, at ages 95 and 100, taking into account all available social and physical environmental factors, that is, “what they get”, in six different cultural settings: Denmark, France (South), Japan (Tokyo and Okinawa), Switzerland and Sweden. These countries have been selected for their low mortality level, the quality of their population and mortality data [4, 6, 30–44], the possibility to draw representative samples of nonagenarians and centenarians, and the existence of research teams working on oldest-old issues [7–10, 20–22, 45–53]. 

## Supplementary Material

The Supplementary Material provides detailed information on the increase in the number of centenarians in each country (Figure A1) and lists the countries having experienced the lowest and the highest mortality levels single age, for each gender and each cohort, through the age mortality trajectory above the age of 50 years (Table A1).

## Figures and Tables

**Table 1:** Number of centenarians (100+) in 2006 and 10-year increase by sex: Denmark, France, Japan, Sweden, and Switzerland.

Number of centenarians (100 and over)				10-Year increase	
Country	Males	Females	Sex-ratio	Males	Females
Japan	3906	23236	5.9	3.0	4.5
France	1532	10941	7.1	2.3	2.0
Switzerland	155	821	5.3	1.9	1.9
Sweden	194	1115	5.7	1.4	1.7
Denmark	99	581	5.9	1.3	1.7

**Table 2:** Chance to survive from 80 to 95 and 100 for cohorts born in 1905 and 1910: Denmark, France, Japan, Sweden, and Switzerland.

	Denmark (= 1)	France	Sweden	Switzerland	Japan
Cohorts born in 1905: Chance to survive from 80 to 100					

Females	1	1.5	1	1	2.4
Males	1	1.6	1.1	1.5	2.6

Cohorts born in 1910: Chance to survive from 80 to 95					

Females	1	1.3	1	1.1	1.7
Males	1	1.3	1	1.2	1.7
