# Supplementary material for: Centenarians Today: New Insights on Selection from the 5-COOP Study
Source: Curr Gerontol Geriatr Res. 2011 Mar 7;2010:120354. doi: 10.1155/2010/120354 (PMC3056212; doi:10.1155/2010/120354)
Supplement: Supplementary file 1 — The Supplementary Material provides detailed information on the increase in the number of centenarians in each country (Figure A1) and lists the countries having experienced the lowest and the highest mortality levels single age, for each gender and each cohort, through the age mortality trajectory above the age of 50 years (Table A1). [file 120354.f1.pdf]

Figure A1: Increase in the number of centenarians (100 and 100+) since 1946*, by sex – the 5-COOP study

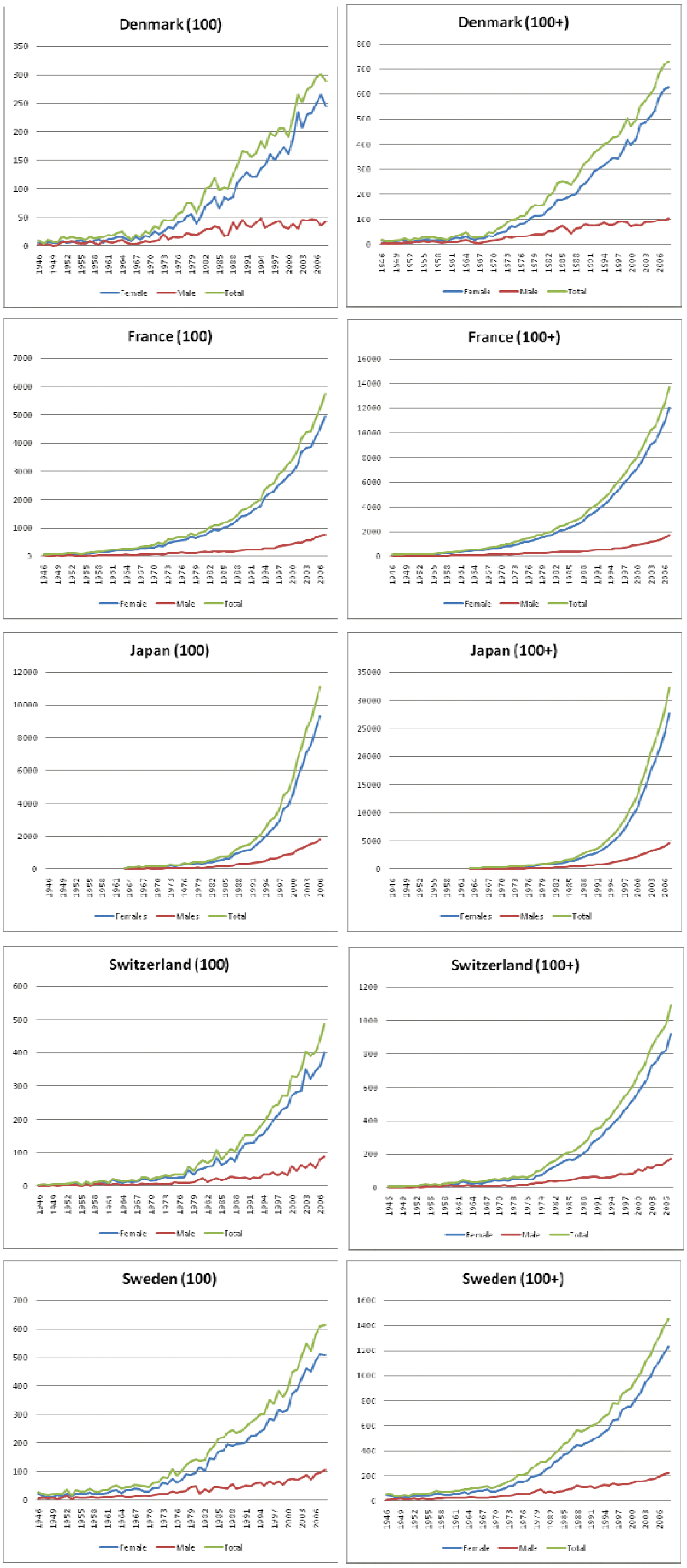

*Since 1963 in Japan - Source of data: Human Mortality Database (HMD) and *Zenkoku koureisha meibo* (for Japan)
