# Supplementary material for: Centenarians Today: New Insights on Selection from the 5-COOP Study
Source: Curr Gerontol Geriatr Res. 2011 Mar 7;2010:120354. doi: 10.1155/2010/120354 (PMC3056212; doi:10.1155/2010/120354)
Supplement: Supplementary file 2 [file 120354.f2.pdf]

Table A1: Countries having experienced the lowest and the highest age mortality levels through the age mortality trajectory above the age of 50 years, by sex and cohort – the 5-COOP study

	Females				Males			
	Cohorts 1905		Cohorts 1910		Cohorts 1905		Cohorts 1910	
**Age**	**Min**	**Max**	**Min**	**Max**	**Min**	**Max**	**Min**	**Max**
**50**	Switzerland	Japan	Switzerland	Japan	Sweden	France	Sweden	France
**51**	Sweden	Japan	Sweden	Japan	Denmark	France	Sweden	France
**52**	Sweden	Japan	Sweden	Japan	Sweden	France	Sweden	France
**53**	Sweden	Japan	Sweden	Japan	Sweden	France	Sweden	France
**54**	Sweden	Japan	Sweden	Japan	Sweden	France	Sweden	France
**55**	Sweden	Japan	Sweden	Japan	Sweden	Japan	Sweden	France
**56**	Sweden	Japan	Sweden	Denmark	Sweden	France	Sweden	France
**57**	Sweden	Japan	Sweden	Japan	Sweden	France	Sweden	France
**58**	Sweden	Japan	Switzerland	Japan	Sweden	France	Sweden	France
**59**	Sweden	Japan	Sweden	Japan	Sweden	France	Sweden	France
**60**	Sweden	Japan	Sweden	Denmark	Sweden	France	Sweden	France
**61**	Sweden	Japan	Sweden	Japan	Sweden	France	Sweden	France
**62**	Sweden	Japan	Sweden	Denmark	Sweden	France	Sweden	France
**63**	Sweden	Japan	France	Denmark	Sweden	France	Sweden	France
**64**	Sweden	Japan	Switzerland	Japan	Sweden	France	Sweden	France
**65**	Sweden	Japan	Switzerland	Denmark	Sweden	France	Japan	France
**66**	Sweden	Japan	Switzerland	Denmark	Sweden	France	Japan	Denmark
**67**	Switzerland	Denmark	Switzerland	Denmark	Sweden	France	Japan	France
**68**	Switzerland	Japan	Switzerland	Denmark	Sweden	France	Japan	Denmark
**69**	Switzerland	Japan	Switzerland	Denmark	Japan	France	Japan	Denmark
**70**	Switzerland	Denmark	Switzerland	Denmark	Japan	France	Japan	Denmark
**71**	Switzerland	Denmark	Switzerland	Denmark	Japan	Denmark	Japan	Denmark
**72**	France	Denmark	Switzerland	Denmark	Japan	Denmark	Japan	Denmark
**73**	Switzerland	Denmark	France	Denmark	Japan	Denmark	Japan	Denmark
**74**	Switzerland	Denmark	Japan	Denmark	Japan	Denmark	Japan	Denmark
**75**	Switzerland	Japan	Japan	Denmark	Switzerland	Denmark	Japan	Denmark
**76**	Switzerland	Sweden	Japan	Denmark	Switzerland	Denmark	Japan	Denmark
**77**	Sweden	Denmark	Japan	Denmark	Japan	Denmark	Japan	Denmark
**78**	Switzerland	Denmark	Japan	Denmark	Japan	Denmark	Japan	Denmark
**79**	Switzerland	Denmark	Japan	Denmark	Japan	Denmark	Japan	Denmark
**80**	Japan	Denmark	France	Denmark	Japan	Denmark	Japan	Denmark
**81**	Switzerland	Sweden	France	Denmark	Japan	Denmark	Japan	Denmark
**82**	France	Denmark	France	Denmark	Switzerland	Denmark	France	Denmark
**83**	Switzerland	Denmark	Japan	Denmark	Japan	Denmark	Japan	Denmark
**84**	Japan	Denmark	France	Denmark	Japan	Sweden	France	Denmark
**85**	France	Denmark	Japan	Denmark	France	Sweden	Japan	Denmark
**86**	France	Denmark	Japan	Denmark	France	Denmark	Japan	Denmark
**87**	France	Denmark	Japan	Denmark	Switzerland	Denmark	Japan	Sweden
**88**	Japan	Denmark	Japan	Denmark	Japan	Denmark	Japan	Denmark
**89**	France	Switzerland	Japan	Switzerland	France	Denmark	Japan	Denmark
**90**	Japan	Denmark	Japan	Denmark	Japan	Denmark	Japan	Denmark
**91**	Japan	Denmark	Japan	Sweden	Japan	Denmark	Japan	Sweden
**92**	Japan	Denmark	Japan	Sweden	Japan	Denmark	Japan	Denmark
**93**	Japan	Sweden	Japan	Denmark	Japan	Switzerland	Japan	Sweden
**94**	Japan	Switzerland	Japan	Sweden	Japan	Denmark	Japan	Sweden
**95**	Japan	Sweden	Japan	Sweden	Japan	Sweden	Japan	Denmark
**96**	Japan	Sweden	:	:	Japan	Sweden	:	:
**97**	Japan	Sweden	:	:	Japan	Switzerland	:	:
**98**	Japan	Denmark	:	:	Denmark	Sweden	:	:
**99**	Japan	Switzerland	:	:	Switzerland	Denmark	:	:
**100**	Japan	Denmark	:	:	Sweden	Switzerland	:	:
